# *In vivo* evaluation of insect wax for hair growth potential

**DOI:** 10.1371/journal.pone.0192612

**Published:** 2018-02-13

**Authors:** Jinju Ma, Liyi Ma, Zhongquan Zhang, Kai Li, Youqiong Wang, Xiaoming Chen, Hong Zhang

**Affiliations:** Research Institute of Resources Insects, Chinese Academy of Forestry, Kunming, Yunnan, China; China Agricultural University, CHINA

## Abstract

Insect wax is secreted by *Ericerus pela* Chavanness. It has been traditionally used to treat hair loss in China, but few reports have been published on the hair growth-promoting effect of insect wax. In this work, we examined the hair growth-promoting effects of insect wax on model animals. Different concentrations of insect wax were topically applied to the denuded backs of mice, and 5% minoxidil was applied topically as a positive control. We found that insect wax significantly promoted hair growth in a dose-dependent manner, 45% and 30% insect wax both induced hair to regrow, while less visible hair growth was observed in blank controls on the 16^th^ day. The experimental areas treated with 45% and 30% insect wax exhibited significant differences in hair scores compared to blank controls, and hair lengths in the 45% and 30% insect wax group was significantly longer than in blank controls on the 16^th^ and 20^th^ days. There were no new hair follicles forming in the treated areas, and the hair follicles were prematurely converted to the anagen phase from the telogen phase in experimental areas treated with 45% and 30% insect wax. Both 45% and 30% insect wax upregulated vascular endothelial growth factor expression. The results indicated that 45% and 30% insect wax showed hair growth-promoting potential approximately as potent as 5% minoxidil by inducing the premature conversion of telogen-to-anagen and by prolonging the mature anagen phase rather than increasing the number of hair follicles, which was likely related to the upregulation of VEGF expression. The dissociative policosanol in insect wax was considered the key ingredient most likely responsible for the hair growth promoting potential.

## Introduction

Hair growth is a complex and cyclically controlled process characterized by a finite period of hair fiber production (anagen), a brief regression phase (catagen), and a resting period (telogen) [1–4]. The hair follicles are remodeled during the cyclical periods [1]. A common disorder of hair growth is alopecia, a generic term for hair loss resulting in a diminution of visible hair [5–7]. Hair loss profoundly impacts social interactions and the psychological well-being of an increasing number of men and women [8–10], and thus the consumer cosmetics and pharmaceutics market for hair re-growth and the protection of hair loss have markedly grown in recent decades. Minoxidil and finasteride are widely used to treat hair loss, but finasteride is associated with adverse sexual effects [11]. Minoxidil is administered topically as a 4–5% solution for the treatment of alopecia but has adverse systemic effects such as anorexia and myocardial infarction [12]. Recently, natural products and plant extracts that can promote hair growth have been widely used in the hair care industry [13–16].

As a natural product, insect wax is secreted by male *Ericerus pela* Chavanness, which live mainly on Chinese privet (*Ligustrum lucidum*) and Chinese ash (*Fraxinus chinensis*) which are widely distributed in most parts of China, Japan and the Korean peninsula from the subtropics to temperate regions [17, 18]. Insect wax is lustrous and free of pollutants, with high melting point and stable chemical properties. With the safe and non-toxic quality, insect wax has been widely used as candle production materials, forming, polishing and coating agents in food science and industry [18–20]. It is well maintaining for fragrances and nutrients of fruits and preventing bacterial for better storage [21]. The application of insect wax has expanded to the pharmaceutical, chemical and cosmetic industries [18, 19, 22, 23]. Hou and Zheng reported that insect wax could promote the granulation tissue proliferation, in addition, it is conducive to hemostasis and relieving pains [21, 24]. The main components of insect wax have been well defined. They include large amounts of wax esters consisting of monobasic saturated fatty alcohols (C26-C30) and monobasic saturated fatty acids (C26-C30), which account for approximately 93–95%. Moreover, insect wax also contains a small amount of dissociative policosanol (1.0–1.5%) which are physiologically active for plants and animals, for example, the octacosanol can strengthen the transport of oxygen and promote skin blood circulation and improve skin activity and prevent eczema and acne after applied in cosmetics [24–28]. In China, insect wax has been used to treat hair loss in folk prescriptions. Ancient texts reported that insect wax promoted hair growth over time when administered topically to the head where hair loss occurred [29]. However, there has been little reported on how insect wax leads to a hair growth-promoting effect. Hence, the present study was performed to evaluate the hair growth-promoting effects of insect wax *in vivo* and to explore the key ingredient most likely responsible for the hair growth-promoting potential.

## Materials and methods

### Materials

Insect wax was supplied by the Emei Insect Wax Institute. Minoxidil tincture (5%, MANDI, ZHEJIANG WAN SHENG PHARMACEUTICAL CO. LTD., ZHEJIANG, CHINA) and camellia oil (Golden Dragon Fish, food grade, WILMAR INTERNATIONAL CO. LTD., SHANGHAI, CHINA) were also purchased, as well as, a mouse vascular endothelial growth factor (VEGF) ELISA Kit (R & D System, R & D SYSTEM BIO-TECHNE CHINA CO. LTD., SHANGHAI, CHINA) and RIPA lysis and extraction buffer (ThermoFisher SCIENTIFIC, SHANGHAI, CHINA). A microtome (RM2126RT, Leica, Wetzlar, Germany), optical microscope (E800, Nikon, Tokyo, Japan) and Vernier caliper (IP54, CHENGDU CHENGLIANG TOOLS GROUP CO., LTD, CHENGDU, CHINA) were used in experiments.

**Fig 1:**
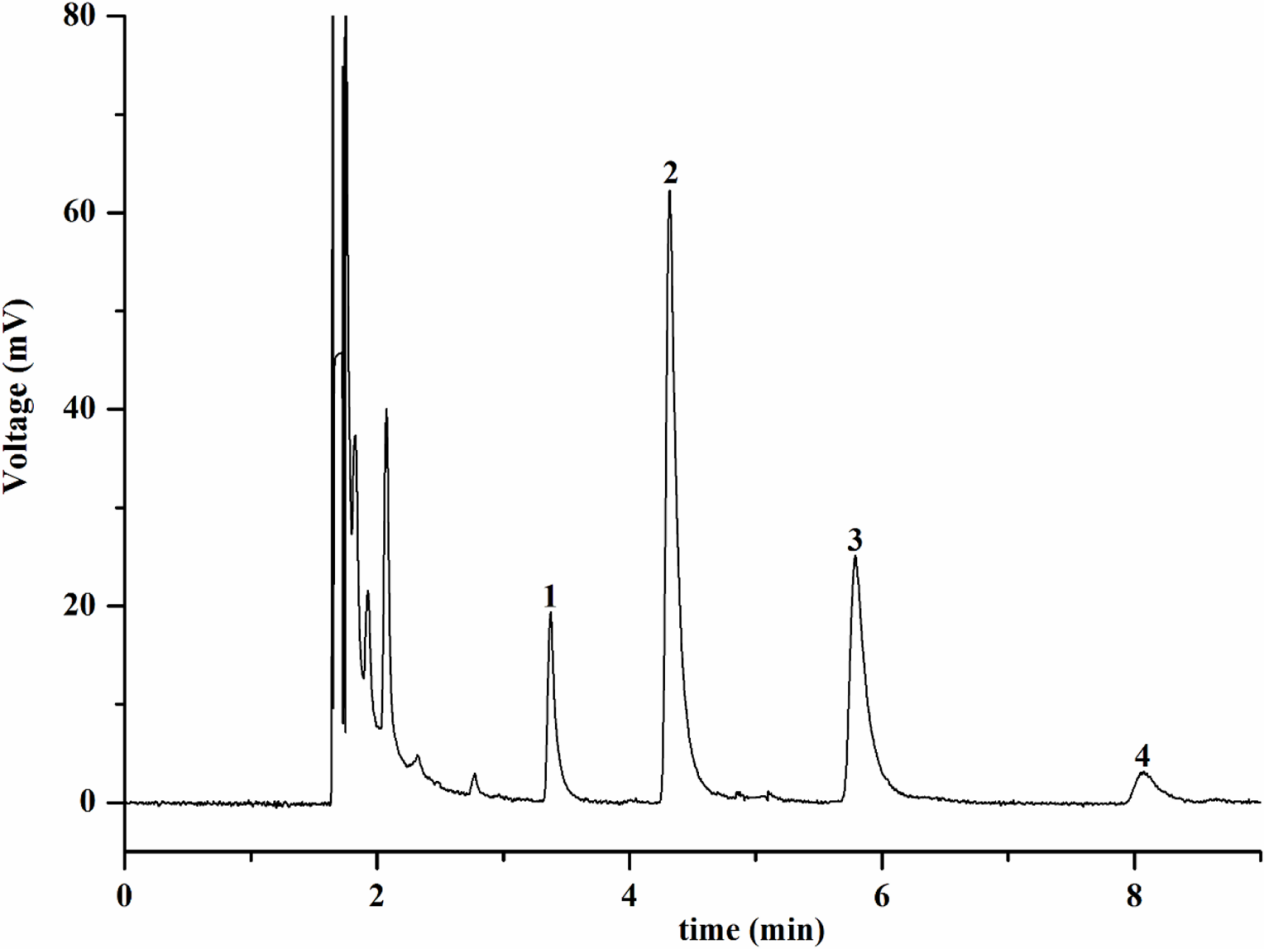
GC chromatogram of policosanol. Policosanol was analyzed by GC-FID using 1:10 split ratio of nitrogen carrier gas injection at 300°C. Peaks: 1, tetracosanol; 2, hexacosanol; 3, octacosanol; 4, triacontanol.

Policosanol was prepared from insect wax in the lab [30]. The composition of policosanol was analyzed by GC-FID using internal standards. The GC chromatogram of policosanol was displayed in Fig 1. The main components of policosanol were tetracosanol, hexacosanol, octacosanol and triacontanol, at contents of 5.2%, 56.0%, 32.0%, 4.0%, respectively.

### Animals

Six-week-old Kunming (KM) mice (animal permit number: 3cdk (chuan) 2013–24, Lot: 0015129) were supplied by Chengdu Dossy Experimental Animals Co., Ltd. (Chengdu, China). The mice were placed in cages and kept in standard environmental conditions controlled under light/dark cycles 12 h/12 h to adapt for one week with standard food and water *ad libitum*. The experimental protocol was approved by the Ethics Committee of the Research Institute of Insect Resources of the Chinese Academy of Forestry (Kunming, China) and the animal experiments were performed in accordance with the guide for Care and Use of Experimental Animals.

### Preparation of ointments

Insect wax was dissolved in camellia oil, which was incubated in a water bath at 85°C to produce the insect wax ointments. The ointments that contained 10%, 30% and 45% insect wax were further used to evaluate the potential hair-growth promoting efficacies *in vivo* based on the results of preliminary experiments. Policosanol was dissolved in camellia oil (0.5%) to study whether dissociative policosanol in insect wax was the key ingredient to promote hair growth. Camellia oil and minoxidil tincture (5%) were used as the negative and positive controls, respectively.

### Assessment of insect wax and policosanol effects on hair growth *in vivo*

One hundred and twenty healthy six-week-old KM mice, half male and half female, were obtained and placed in cages with standard environmental conditions to adapt for one week with food and water ad libitum in October. The KM mice were divided into 6 groups randomly, and the hairs on the back area of the bilateral symmetry of the spine in all mice were removed with 6% sodium sulfide at 7 weeks of age, at which time all hair follicles were synchronized in the telogen phase [31]. A circle around the denuded area was marked with picric acid. These mice were treated with 10%, 30%, or 45% insect wax ointment, camellia oil, minoxidil tincture (5%), or 0.5% policosanol. Specifically, 0.20 g of insect wax ointment, camellia oil (0.20 mL), minoxidil tincture (5%, 0.20 mL) or 0.5% policosanol (0.18 g) were topically applied onto either side of the denuded area of the bilateral symmetry of the spines of mice as the experimental area in a separated group twice a day, and the other side of the denuded area received no treatment as a blank control area. This treatment was continued for 35 days, during which time the hair re-growth activities were observed visually and recorded in photographs every day. The effective rates of the 6 groups were statistically analyzed. The experimental samples were considered to effectively promote hair growth when the hairs in experimental areas regrew earlier than that in the corresponding blank control areas; these data were the basis of effective rates statistics (the effective rate = the number of effective mice / the total number of mice). The hair re-growth efficacies were also evaluated with the following hair growth score: score 0, no hair growth observed; score 1, shallow-hair covered experimental area observed; score 2, length and density of re-grown hairs were half of the original hairs; score 3, length and density of re-grown hairs were the same as the original hairs. The score was calculated according to the area ratio of the re-growth hairs in the experimental or blank control area if the hair grew irregularly [32].

### Hair length determination

The hairs in the experimental and blank control area of each mouse from each group were plucked randomly on the 16^th^, 20^th^, 30^th^ day. The lengths of 30 hairs were measured manually with Vernier caliper, and the average length was determined and expressed as the mean length ± S. D.

### Histological studies

The dorsal skin in the experimental and blank control areas was excised after the KM mice were treated daily with insect wax ointments for 0, 12 and 20 days. The skin tissues were fixed in 4% paraformaldehyde solution and embedded in paraffin wax. The embedded skin tissues were cut to 6 μm-thick slices using the microtome, and the sliced tissues were stained with hematoxylin–eosin and observed under an optical microscope.

### GC-FID analyses of policosanol

The policosanol was analyzed by GC-FID equipped with a BD-5 capillary column (30 m × 0.25 mm i.d., film thickness 0.25 μm) using a 1:10 split ratio of nitrogen carrier gas at a column flow of 1.7 mL/min. The injector and detector temperatures were set at 300°C. The injection volume was 2.0 μL. The temperature of the column was set at 290°C.

### Effect of insect wax and policosanol on expression of vascular endothelial growth factor (VEGF)

The effects of 45%, 30%, and 10% insect wax, camellia oil, minoxidil tincture (5%) and 0.5% policosanol on the expression of VEGF in skin was measured by ELISA. The dorsal skin of mice in experimental and blank control areas in different groups on the 20^th^ day was excised (0.1000 g) and cleaved with RIPA lysis and extraction buffer (200 uL) after grinding with liquid nitrogen, and then was dropped into the wells of the ELISA plate (100 uL / well). The expression of VEGF was measured according to the description in the mouse vascular endothelial growth factor ELISA Kit (R & D System), and the standard curve was constructed using the following concentrations: 0, 15.62, 31.25, 62.50, 125.00, 250.00, 500.00, 1000.00 pg/ml. The results were expressed as pg / g tissue.

## Results and discussions

### Effect of insect wax on hair growth activity *in vivo*

**Fig 2:**
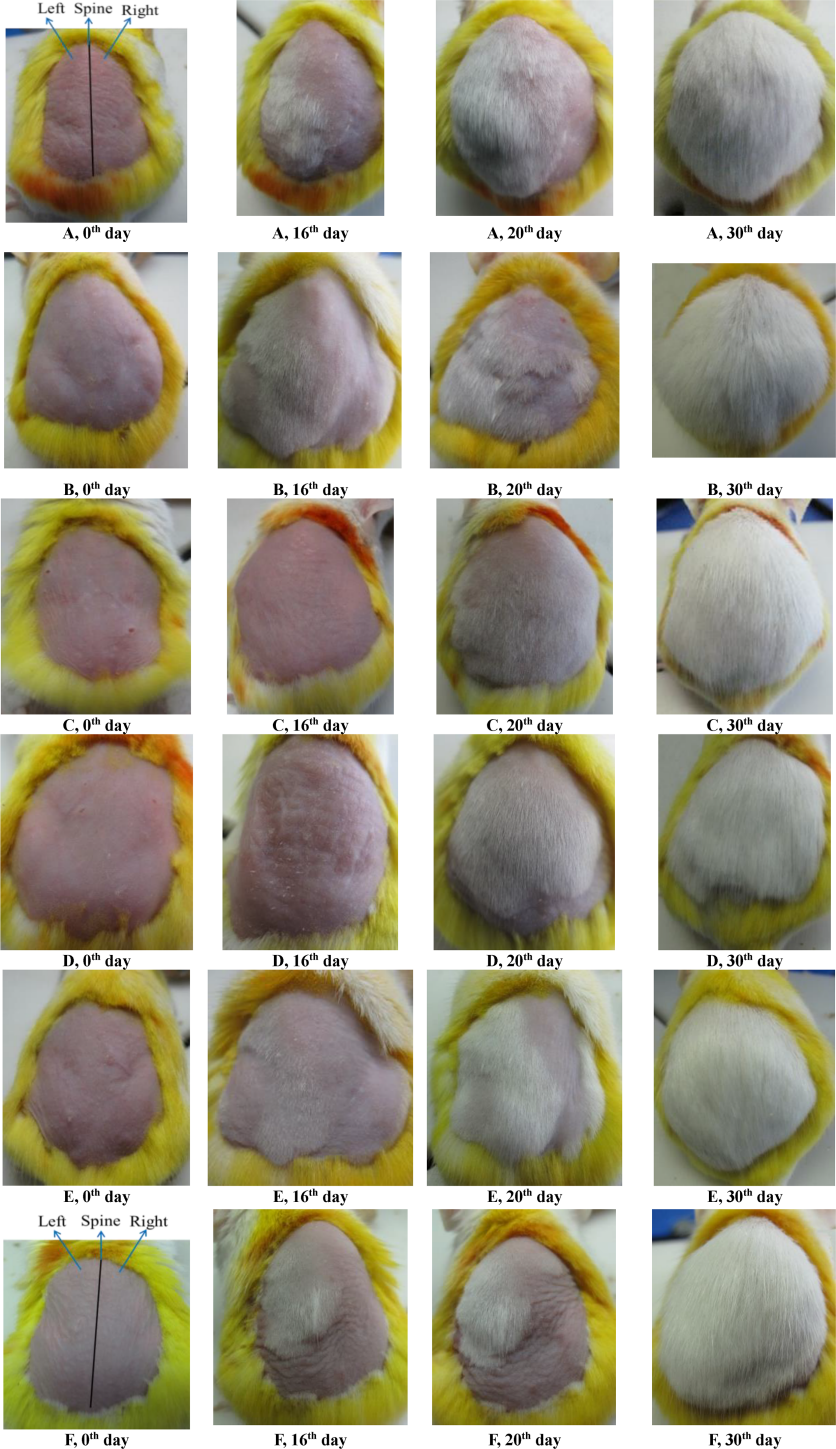
*In vivo* hair growth-promoting efficacies on the 0^th^, 16^th^, 20^th^ and 30^th^ day in different groups. 45% insect wax (A), 30% insect wax (B), 10% insect wax (C), camellia oil (D), 5% minoxidil tincture (E) and 0.5% policosanol (F). Left: experimental area, right: blank control area.

**Fig 3:**
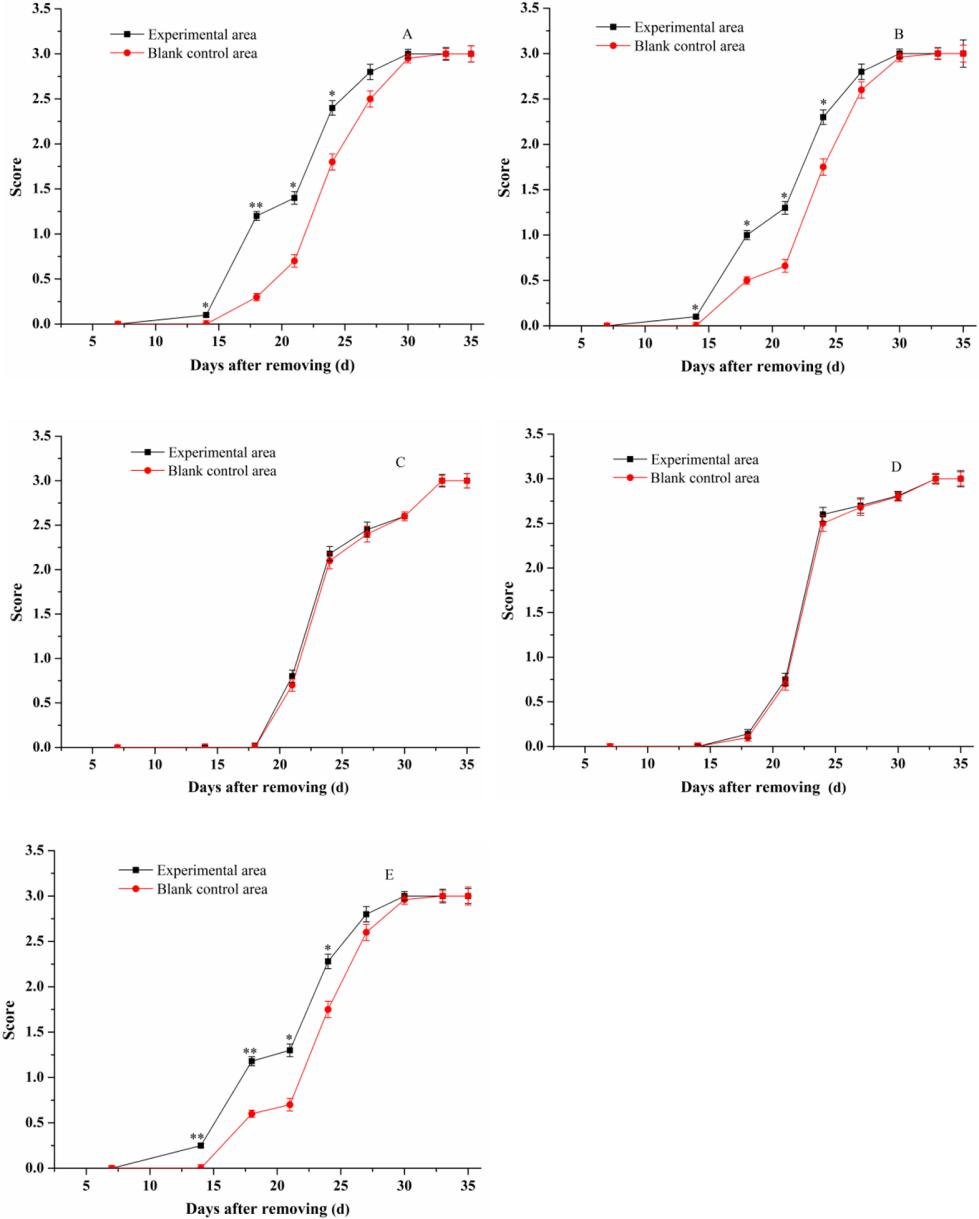
Hair growth-promoting scores in different groups. 45% insect wax (A), 30% insect wax (B), 10% insect wax (C), camellia oil (D) and 5% minoxidil tincture (E). * P < 0.05, ** P < 0.01, compared with blank control areas.

Depilation synchronized hair cycles, such that there were no temporal differences between individuals or spatial differences between the caudal and cranial regions of the back skin. The prepared insect wax ointments, camellia oil and minoxidil tincture were applied to the depilated KM mice for 35 days. The differences in hair growth promotion between treatment and non-treatment areas in the animals were observed and recorded in photographs. During the application period, differences in the average body weight of mice were not observed. Fig 2A–2E shows the hair growth-promoting efficacy of 45%, 30%, 10% insect wax, camellia oil and 5% minoxidil tincture. The backs of the mice were hairless on the 0^th^ day (hair growth score: 0, Fig 3). On the 16^th^ day, we observed that 45% and 30% insect wax induced the hair to regrow in the denuded skin of the experimental areas, with less visible hair growth in the corresponding blank control areas. The whole denuded skin in experimental areas had been covered by re-growth hairs when treated with 45% and 30% insect wax on the 20^th^ day, while the re-growth hairs in the corresponding blank control areas of above two groups were very sparse on the 20^th^ day (Fig 2A, Fig 2B). This clearly indicates that 45% and 30% insect wax both promoted KM mouse hair regrowth after removal. In addition, the experimental areas in the 45% and 30% insect wax groups both exhibited significant differences in hair scores on the 14^th^, 18^th^, 21^st^, 24^th^ day compared to their corresponding blank control areas (Fig 3A, Fig 3B), in good agreement with the appearance in photos. However, further improvements in growth promotion by 10% insect wax and camellia oil were not visually apparent (Fig 2C, Fig 2D) and neither groups' hair growth scores exhibited significant differences between the experimental areas and the corresponding blank control areas during the application period (Fig 3C, Fig 3D). This means that 10% insect wax and camellia oil almost had no efficacy to promote hair-growth. There were also significant differences in hair scores between the experimental area and the corresponding blank control area in the 5% minoxidil tincture group (minoxidil is an OTC drug for hair re-growth) (Fig 3E). Moreover, Table 1A–1E displayed the effective rates of 45%, 30%, 10% insect wax, camellia oil and 5% minoxidil tincture on hair re-growth after removal in KM mice. The hair growth-promoting efficacy of 10% insect wax was almost the same as for camellia oil, and both effective rates were 10%, which meant that 10% insect wax and camellia oil had no efficacy to promote hair-growth. The effective rates of 45%, 30% insect wax and 5% minoxidil tincture were significantly higher (P < 0.01) than for 10% insect wax and camellia oil (Table 1). Insect wax was evaluated for its promoting of hair growth by Zhan-di Wang, using an androgenetic alopecia animal model administering testosterone propionate [33]. Interestingly, model animals treated with 10% insect wax dissolved in a vehicle (absolute ethyl alcohol/1,2-propylene glycol, 80:20) showed less hair loss in Wang’s study, but 10% insect wax dissolved in camellia oil was not effective in this study. Absolute ethyl alcohol and 1,2-propylene glycol had better permeability and hydrophilicity than camellia oil, leading to enhanced permeation and easier crossing cells into the target site of hair follicle. In addition, this work used physical methods to change the growth stage of hair follicles, and the model animals were subcutaneously administered testosterone propionate in Wang’s study [33], which led the hair follicles to be in fundamentally different states. Thus, there was no comparability in the dosage in two different animal models.

**Table 1:** Effects of 45% (A), 30% (B), 10% insect wax (C), camellia oil (D) and 5% minoxidil tincture (E) on hair regrowth after removal in KM mice.

Groups	Number of experimental KM mice	Effective rate/ %
A, 45% insect wax	20	78.8 ^a^
B, 30% insect wax	20	80.0 ^a^
C, 10% insect wax	20	10.0 ^b^
D, camellia oil	20	10.0 ^b^
E, 5% minoxidil tincture	20	80.0 ^a^
F, 0.5% policosanol	20	78.6 ^a^

Note: Same letters in table differentiated no significantly, different letters differentiated very significantly ((P < 0.01).

### Hair length determination

**Fig 4:**
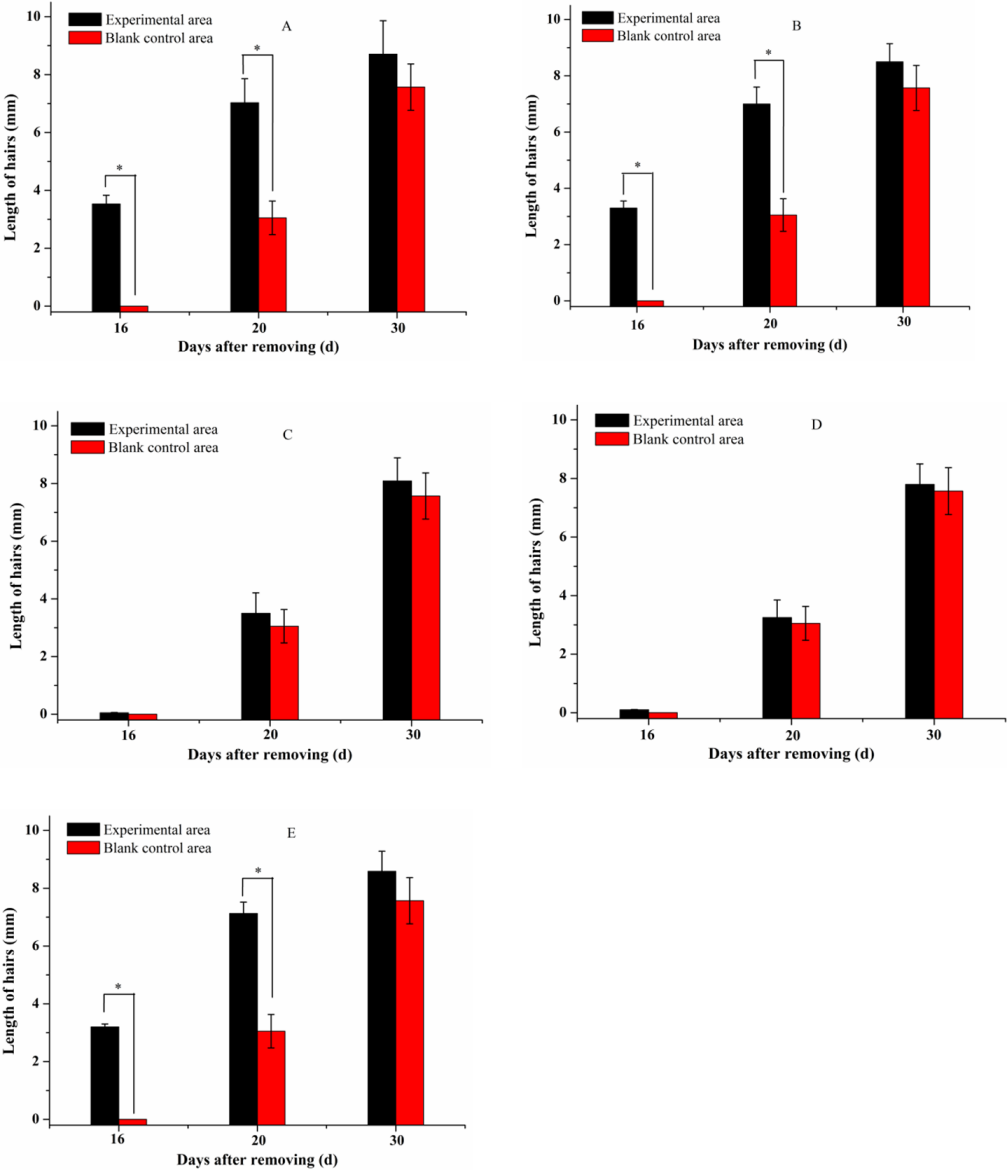
Effect of different treatments on hair length. 45% insect wax (A), 30% insect wax (B), 10% insect wax (C), camellia oil (D) and 5% minoxidil tincture (E). * P < 0.01, compared with corresponding blank control, respectively (n = 30 hairs).

The visible hair shaft was observed from the denuded area at the end of the 2nd week and the length of the hair began to increase until the end of the treatment course. The length of randomly plucked hairs was measured at different time intervals (16^th^, 20^th^ and 30^th^ day) after topical application of 45%, 30%, 10% insect wax, camellia oil and 5% minoxidil tincture. Data represented the mean ± standard deviation (S.D.) of three independent experiments (Fig 4). One-way ANOVA was used for comparison of multiple group means. As shown in Fig 3, the length of hairs in 45% and 30% insect wax treated groups was significantly longer than the corresponding blank control (p-value < 0.01) on the 16^th^ and 20^th^ days (Fig 4A, Fig 4B). This may be due to the premature switching of follicles from the telogen to the anagen phase of the hair growth cycle, as observed by Babu and Singh [34, 35]. Uno and Kurata reported that topical application of fuzzy rat with minoxidil, diazoxide and copper peptide produced conversion of short vellus hairs to long terminal hairs and an enlargement of follicular size with prolongation of the anagen phase by enhancing the rate of cell proliferation [36]. There were no significant differences in the length of hairs with 10% insect wax—or—camellia oil—treated groups and the corresponding blank control areas (Fig 4C, Fig 4D). Thus, 45%, 30% insect wax and 5% minoxidil tincture could promote the hair growth of KM mice. However, 10% insect wax and camellia oil almost had no hair-growth- promoting effect. It could be initially inferred that 45% and 30% insect wax induce hairs in denuded areas of mice to regrow prematurely, and there were no obvious differences in the efficacies between the 45%, 30% insect wax and minoxidil tincture (5%).

### Histological observation of hair follicles

**Fig 5:**
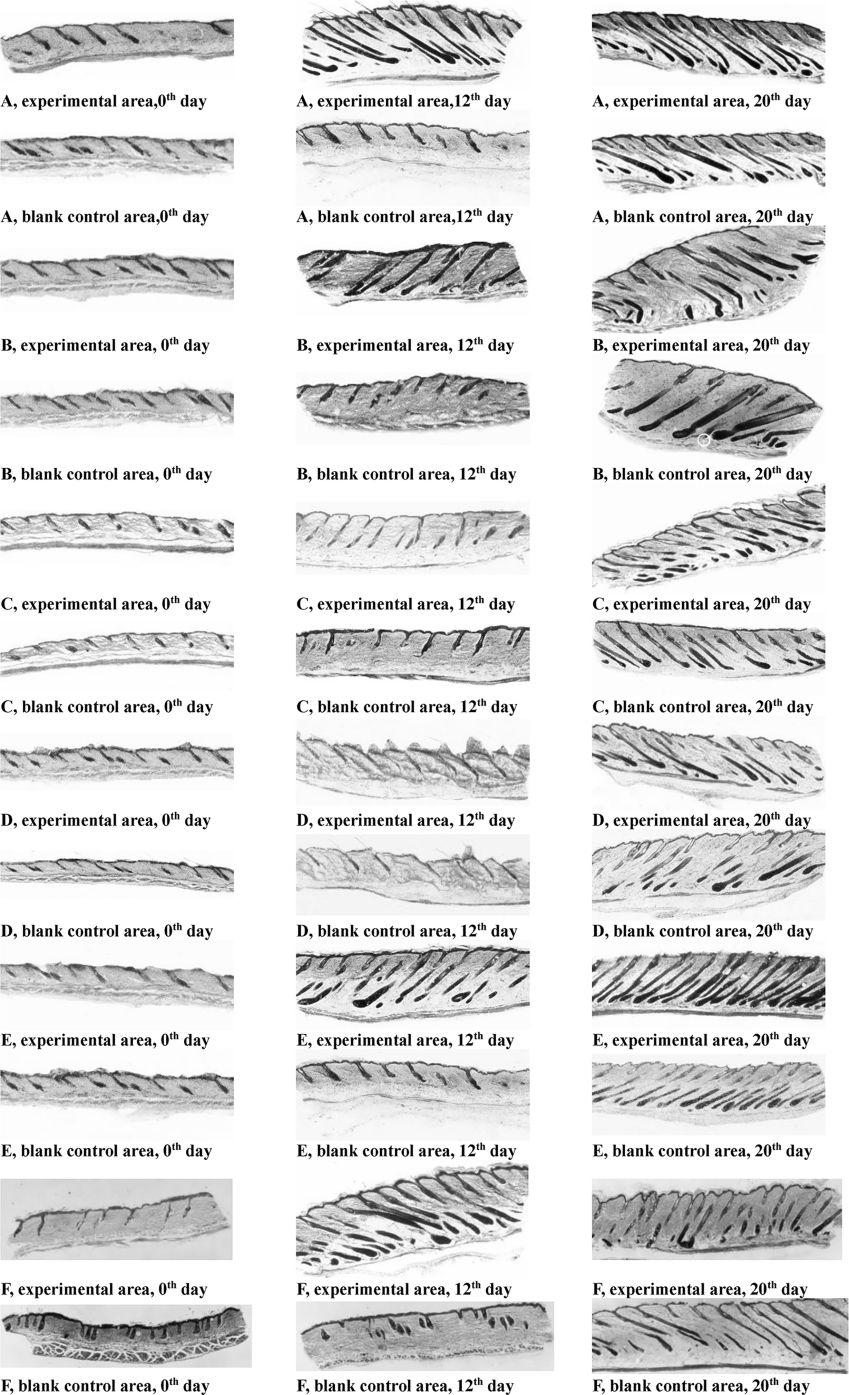
Optical microscope photos of skins in different groups on the 0^th^, 12^th^ and 20^th^ day. 45% insect wax (A), 30% insect wax (B), 10% insect wax (C), camellia oil (D), 5% minoxidil tincture (E) and 0.5% policosanol (F) (original magnification was × 100).

**Fig 6:**
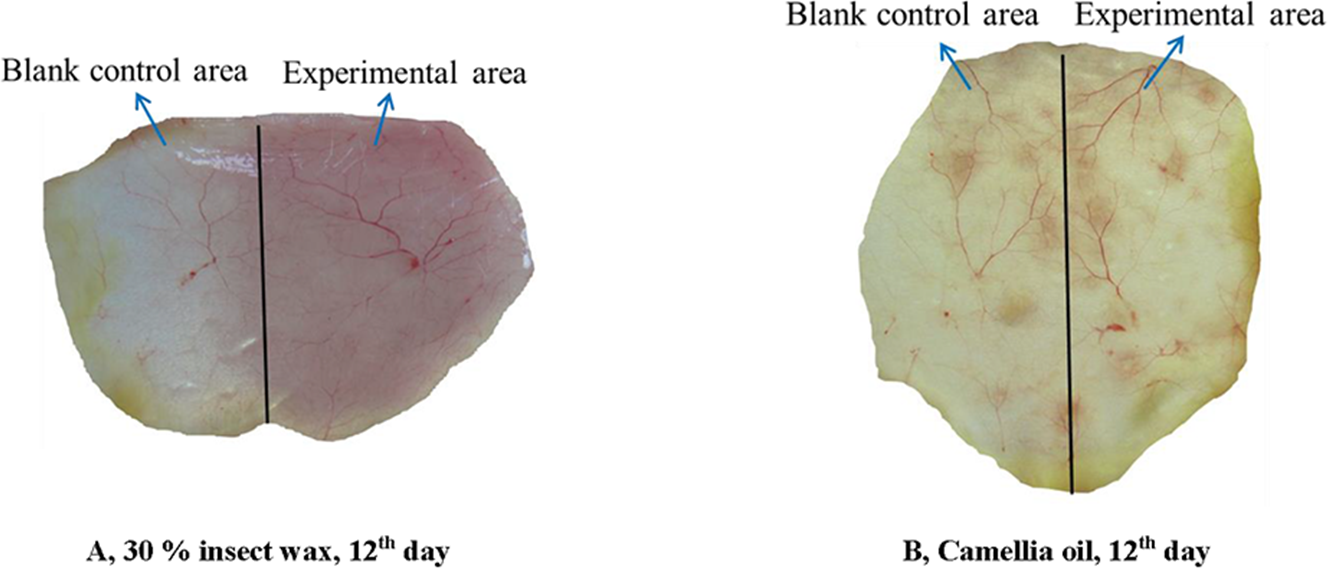
Tissue appearance of reverse side of skin on the 12^th^ day after application of 30% insect wax and camellia oil onto depilated KM mice.

**Fig 7:**
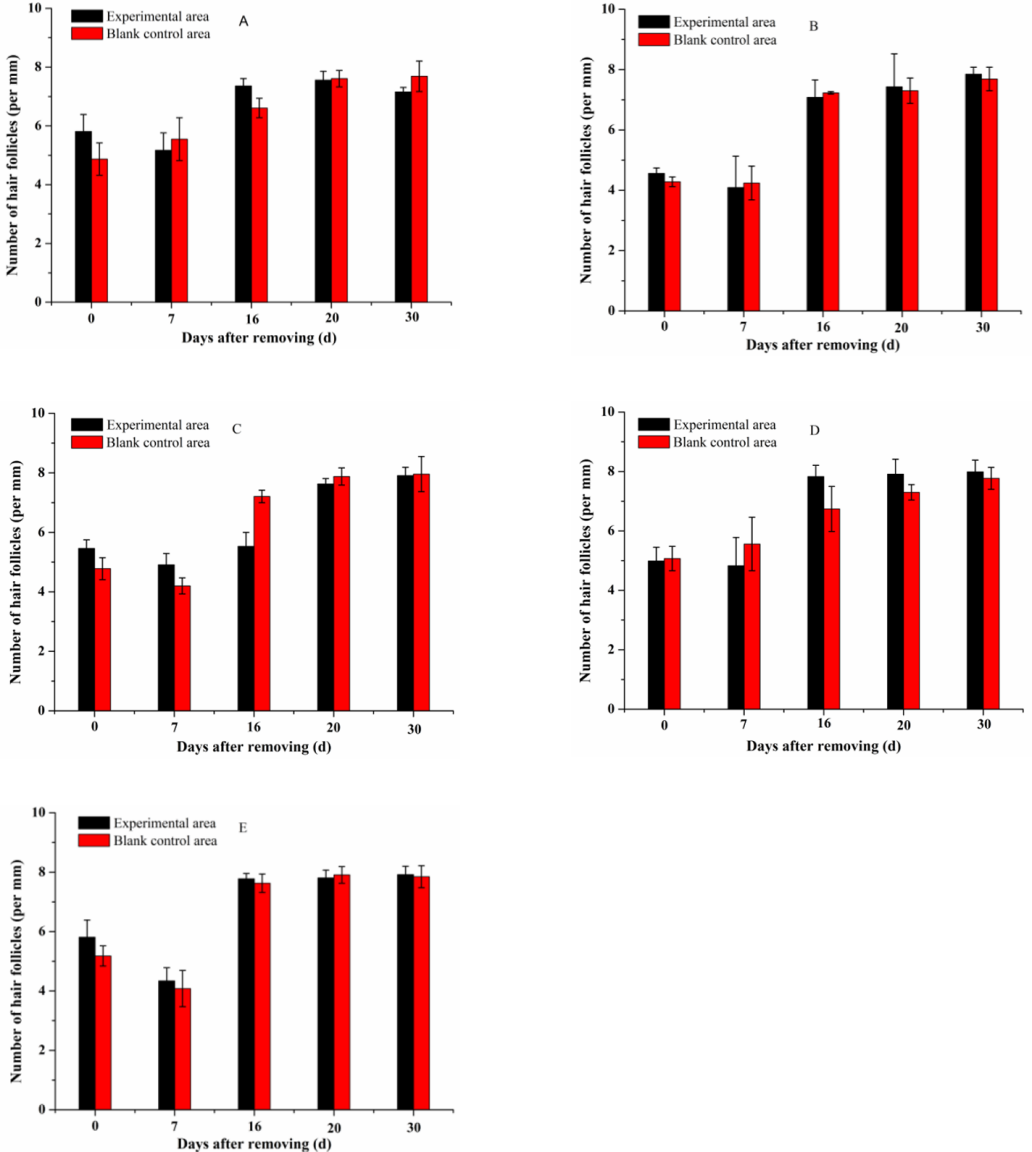
Number of hair follicles in experimental and corresponding blank control areas in different groups. 45% insect wax (A), 30% insect wax (B), 10% insect wax (C), camellia oil (D) and 5% minoxidil tincture (E). Data were statistically analyzed on the 0^th^, 7^th^, 16^th^, 20^th^ and 30^th^ days.

Fig 5 displays optical microscopic photos of hematoxylin-eosin stained slices of skins obtained on the 0^th^, 12^th^ and 20^th^ day from the backs of mice daily treated with 45%, 30%, or 10% insect wax, camellia oil, 5% minoxidil tincture or the corresponding blank control of each group. The hair follicles in experimental areas and the corresponding blank control areas for all groups were in the telogen phase on the 0^th^ day after removing hair (Fig 5A–5E). However, marked differences in the different cyclic phases (anagen and telogen) of hair follicles in treated areas and corresponding blank control areas were observed on the 12^th^ day. The hair follicles in the experimental and the corresponding blank control areas of mice treated with camellia oil were nearly all in the telogen phase on the 12^th^ day, and the hair follicles were believed to be in the anagen phase on the 20^th^ day (Fig 5D). The hair follicles in the experimental and blank control areas of mice treated with 10% insect wax were in the telogen phase on the 12^th^ day and in anagen phase on the 20^th^ day (Fig 5C). The 10% insect wax and camellia oil had almost no hair growth-promoting efficacy. The hair follicles in the experimental areas of mice treated with 45% and 30% insect wax were already in the anagen phase on the 12^th^ day, and the thicker and longer hair follicles were observed on the 20^th^ day. Hair follicles in the corresponding blank control areas of the above two groups were basically still in telogen phase on the 12^th^ day and were in anagen phase on the 20^th^ day (Fig 5A and 5B). Fig 6 illustrated two photographs of the reverse side of back skin flaps on the 12^th^ day after application of 30% insect wax and camellia oil onto the depilated KM mice. It was apparent from Fig 6A that anagen skin in the experimental area treated with 30% insect wax was thickened and a number of blood vessels were newly formed, compared to the corresponding blank control area, which was in the telogen phase. In contrast, there was little difference between the telogen skin in the experimental area treated with camellia oil and the corresponding blank control area (Fig 6B). Based on the above results, it was conceivable that 45% and 30% insect wax may induce hair follicles to prematurely convert to anagen phase from telogen phase and prolong the mature anagen phase in hair follicles. Moreover, mice treated with 5% minoxidil tincture were already in the anagen phase on the 12^th^ day, and healthy hair follicles were found on the 20^th^ day (Fig 5E). As discussed in the previous sections, 45% and 30% insect wax may promote hair growth in denuded areas of healthy KM mice, while 10% insect wax showed almost no hair growth-promoting efficacy, which may be due to too little insect wax. The results of histological observation of hair follicles were in good agreement with the photos. Moreover, the number of hair follicles in experimental areas and the corresponding blank control areas of KM mice daily treated with 45%, 30%, or 10% insect wax, camellia oil and 5% minoxidil tincture on the 0^th^, 7^th^, 16^th^, 20^th^ and 30^th^ day are displayed in Fig 7A–7E. The histological study proved no difference in the number of hair follicles in the treated and blank control groups, with 4–8 hair follicles per mm of the skin. This clearly indicated that no new hair follicles formed in the treated areas. As an OTC drug for hair re-growth, minoxidil is known to dilate blood vessels surrounding hair bulbs and enhance the delivery of oxygen and nutrients to the cells of hair follicles [37]. However, the exact mechanism of the stimulation of hair growth is not known. Similarly, in our study, we observed that hair follicles periodically transformed from telogen to anagen phase in all groups. The 45% and 30% insect wax treatments both induced premature telogen-to-anagen phase conversion. However, the exact mechanism of insect wax on hair growth requires further study.

### Effect of policosanol on hair growth activity in vivo

Insect wax at concentrations of 30% or higher showed hair-growth promoting potential in the above experimental results, and insect wax contains approximately 1.5% dissociative policosanol [24, 25, 38]. Thus, the content of dissociative policosanol in 30% and 45% insect wax was or above 0.5%, in this work. The 10% insect wax treatment did not exhibit any hair growth-promoting efficacy, which may be due to too little content of policosanol. Therefore, the policosanol in insect wax may be the key ingredient most likely responsible for hair growth-promoting potential. The hair growth-promoting efficacy of 0.5% policosanol was studied to verify that the dissociative policosanol in insect wax was the key ingredient to promote hair growth. As seen in Fig 2F, the backs of mice were hairless on the 0^th^ day. The experimental areas treated with 0.5% policosanol were covered by hairs at day 20, but the re-grown hairs in the blank control areas were sparse and invisible even on the 20^th^ day (Fig 2F), indicating that 0.5% policosanol produced significant hair growth-promoting effects in mice. Table 1F displayed the effect of 0.5% policosanol on hair growth. The effective rate of 0.5% policosanol was significantly higher than that of camellia oil (P < 0.01). There was no obvious difference in the effective rates between 0.5% policosanol and 30% insect wax (Table 1), indicating that 0.5% policosanol promoted hair growth. In addition, after topical application of 0.5% policosanol to the backs of KM mice, the premature telogen-to-anagen phase conversion also was induced, based on the histological study (Fig 5F). Hence, the dissociative d policosanol in insect wax was considered the key ingredient to promote hair growth.

### Effect of insect wax and policosanol on expression of VEGF

**Fig 8:**
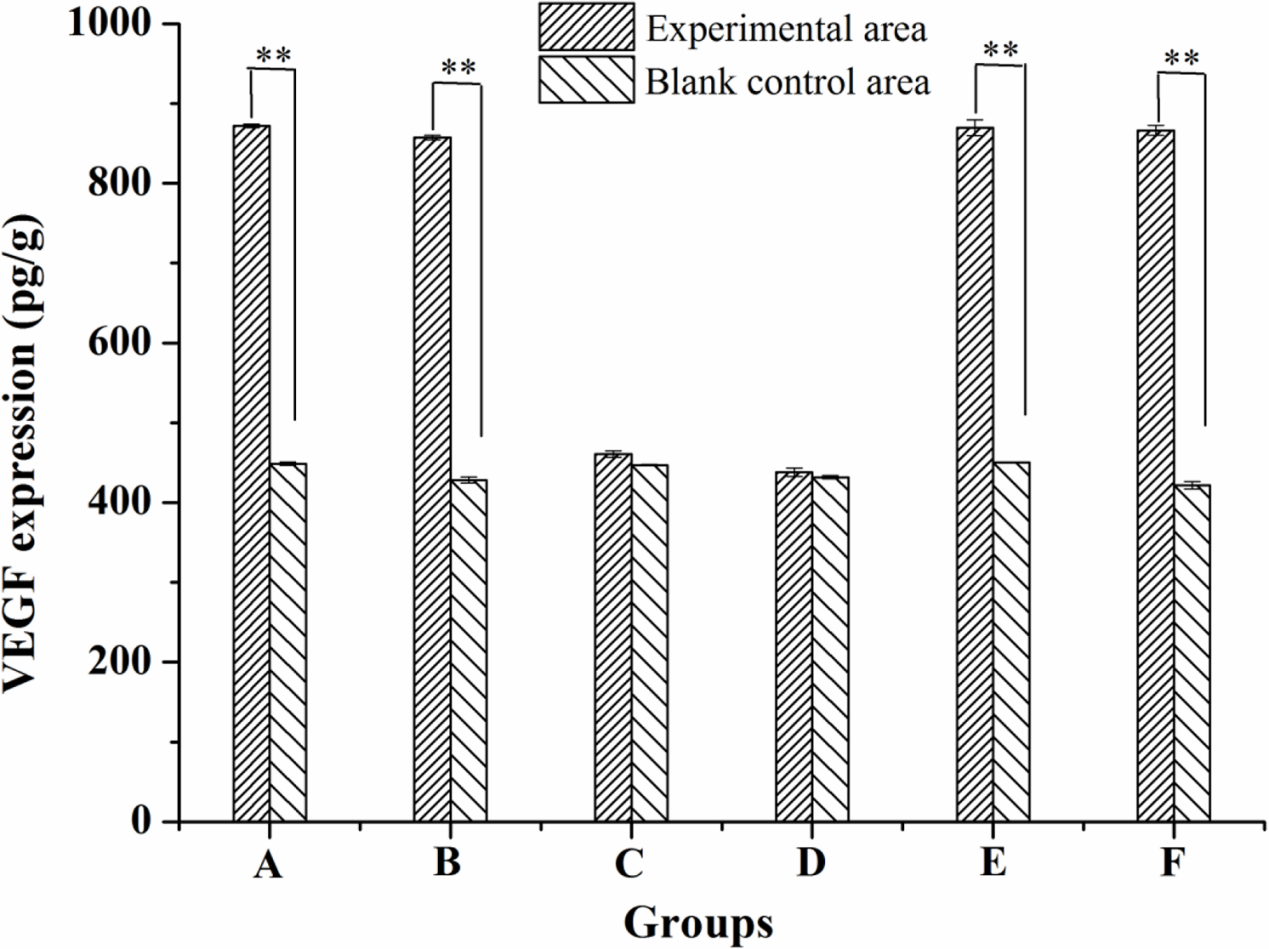
VEGF expression in experimental and corresponding blank control areas in different groups. 45% insect wax (A), 30% insect wax (B), 10% insect wax (C), camellia oil (D), 5% minoxidil tincture (E) and 0.5% policosanol (F), ** P < 0.01, compared to corresponding blank control (n = 3).

As seen in Fig 8, the VEGF expression levels in experimental areas of 45%, 30% insect wax, 5% minoxidil and 0.5% policosanol were significantly higher than the corresponding blank control areas (P < 0.01). There were no significant differences among 45%, 30% insect wax, 5% minoxidil and 0.5% policosanol (P > 0.05). In other words, 45%, 30% insect wax and 0.5% policosanol upregulated the expression of VEGF in mice hair follicles, similar to the 5% minoxidil group. However, there were no differences in the VEGF expression between 45% and 30% insect wax (P > 0.05). VEGF is a growth factor that can stimulate the formation of new blood vessels and reflect the nutrition of hair follicles. The high expression level of VEGF promotes the supply of nutrients to the hair follicle [39, 40], which may contribute to the extension of anagen phase and the premature conversion of telogen-to-anagen and further stimulation of hair growth. Minoxidil has been used extensively for many years to treat androgenic alopecia. Minoxidil can promote the vascularization of hair dermal papilla by upregulating the expression of VEGF in normal human hair follicles [41]. Young Chul Kim reported that *Chamaecyparis obtusa* oil had an excellent growth effect in C57BL/6 mice and significantly improved VEGF expression in skin tissue [42]. Therefore, we speculated that 45% and 30% insect wax and 0.5% policosanol promoted hair growth by upregulating VEGF expression, which may induce the premature telogen-to-anagen phase conversion and prolong the mature anagen phase in hair follicles. Moreover, the experimental phenomena in the 0.5% policosanol group and 30% insect wax displayed similar patterns, which further demonstrated that policosanol in insect wax may be the key ingredient most likely responsible for the hair-growth promoting potential. The therapeutic mechanisms of insect wax will require further study.

## Conclusions

In summary, insect wax at concentrations of 45% and 30% showed excellent hair-growth promoting efficacies, which were approximately as potent as minoxidil tincture (5%). Both 45% and 30% insect wax induced the premature conversion of telogen-to-anagen and prolonged the mature anagen phase in hair follicles rather than increasing the number of hair follicles, which was likely related to upregulation of VEGF expression. The dissociative policosanol in insect wax was considered the key ingredient most likely to be responsible for the hair growth-promoting potential. This work may provide guidance for the application of insect wax as a promising hair growth agent.

## Supporting information

S1 File Graphical abstract. (DOC)

S2 File PONE-D-17-36203 raw data. (XLS)

S3 File PONE-D-17-36203 raw data. (XLS)

S4 File PONE-D-17-36203 raw data. (XLS)

S5 File PONE-D-17-36203 raw data. (XLS)

S6 File PONE-D-17-36203 raw data. (DOC)
