# Supplementary material for: In vivo evaluation of insect wax for hair growth potential
Source: PLoS One. 2018 Feb 13;13(2):e0192612. doi: 10.1371/journal.pone.0192612 (PMC5811011; doi:10.1371/journal.pone.0192612)

**Graphical Abstract**

Title: *In vivo* evaluation of insect wax for hair growth potential

Authors: Jinju Ma, Liyi Ma*, Zhongquan Zhang, Kai Li, Youqiong Wang, Xiaoming Chen, Hong Zhang


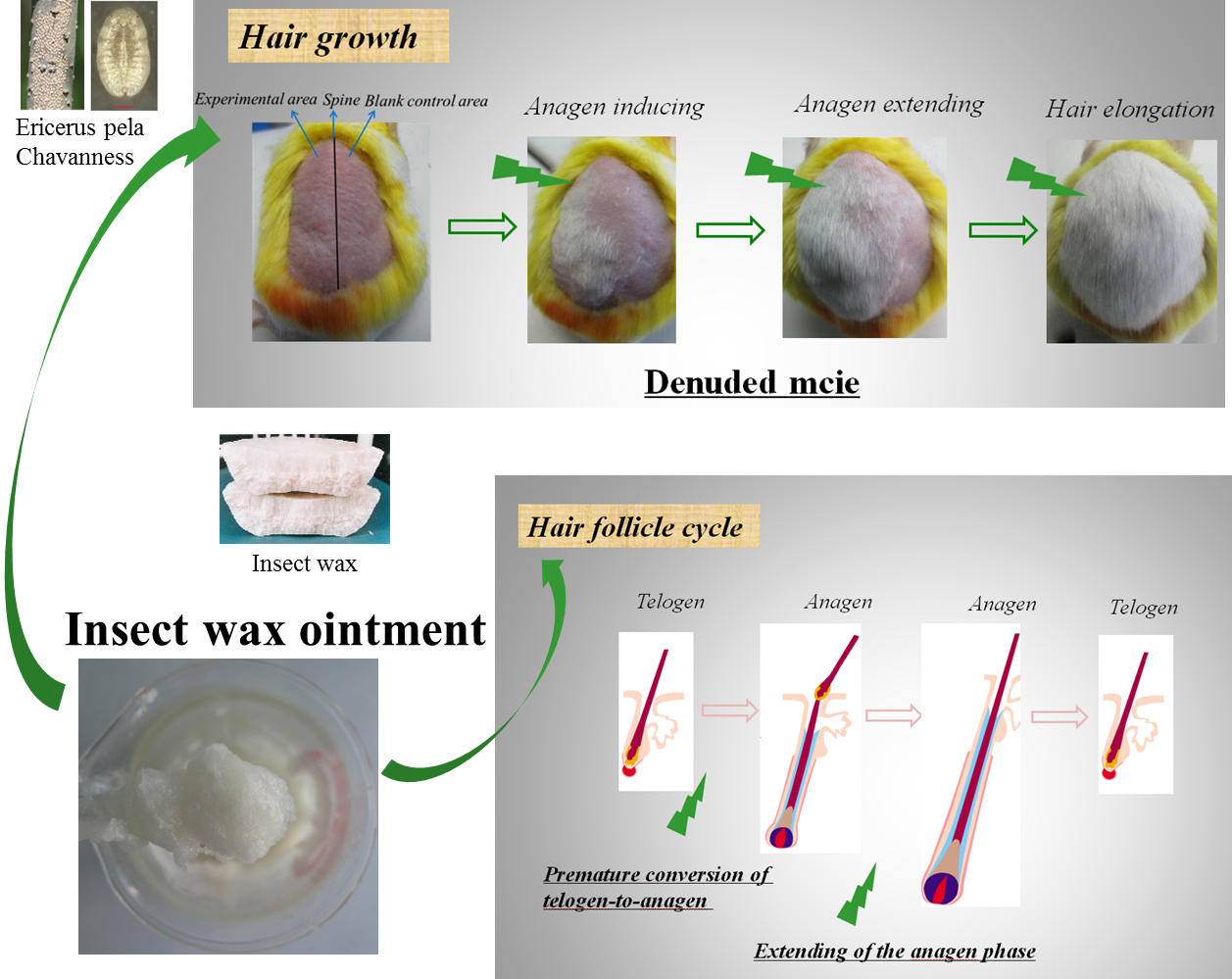

Supplement: S1 File — (DOC) [file pone.0192612.s001.doc]
