# Supplementary figures and images for: In vivo evaluation of insect wax for hair growth potential
Source: PLoS One. 2018 Feb 13;13(2):e0192612. doi: 10.1371/journal.pone.0192612 (PMC5811011; doi:10.1371/journal.pone.0192612)

A sample enzyme linked immunosorbent assay image


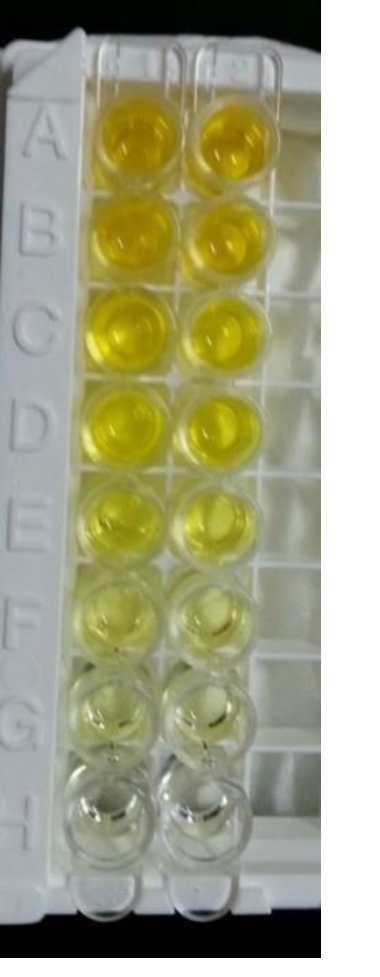

Supplement: S6 File — (DOC) [file pone.0192612.s006.doc]
